# Myths about suicide - validating the Hungarian version of the Literacy of Suicide Scale (H-LOSS) on a community sample

**DOI:** 10.1186/s12889-024-19841-8

**Published:** 2024-08-29

**Authors:** Richard Flach, Robert Fodor, Flora Kettel-Fulop, Peter Osvath, Andras Lang

**Affiliations:** 1 Institute of Psychology, University of Pécs, 6 Ifjusag Street, Pécs, Baranya, 7624 Hungary; 2 Ulster University, Belfast, Northern Ireland UK; 3 Department of Psychiatry and Psychotherapy, Medical School, University of Pecs, Pecs, Hungary

**Keywords:** Literacy of suicide, Suicide prevention, Item response theory, Validity, Public population

## Abstract

**Background:**

Suicide was exceptionally high in Hungary in the last century. According to Eurostat, Hungary ranks second in the EU in death by suicide and was among the few countries where the suicidal tendencies increased in 2020. Primary tasks of suicide prevention programs are to develop suicide literacy and dispel myths and misconceptions about suicide. Therefore, the goal of our research was the Hungarian validation of the 26-item Literacy of Suicide Scale (LOSS). Methods: 749 people (382 female (51.0%), 364 male (48.6%), 3 identify as non-binary or other (0.4%); 4 identifying as transgender (0.5%)) participated in our online cross-sectional survey with a mean age of 32.4 years (SD = 14.5 years). The H-LOSS questionnaire was adapted using the 2PL (two-parameter logistic) model with WLSE (weighted least squares) estimation in item response theory method, similarly to the original English version.

**Results:**

Scale unidimensionality was confirmed. Model fit indices and internal reliability indicators were acceptable. Item infit and outfit values were adequate, item discrimination values were within range, but one item had extremely high and three items had extremely low item difficulty parameters. Few items had differential item functioning by age, gender and own suidice attempt.

**Conclusions:**

The H-LOSS scale deemed to be appropriate for assessing suicide literacy in Hungarian speaking samples.

**Supplementary Information:**

The online version contains supplementary material available at 10.1186/s12889-024-19841-8.

## Introduction

According to the World Health Organisation [1] report 2023, around 700,000 people worldwide committed suicide every year, raising significant public health challenges. In terms of suicide deaths, Hungary ranks high among OECD countries, with traditionally high suicide deaths despite favourable changes [2]. In Hungary, in the last century, the number of committed suicide was extremely high [3], - and Hungary has had one of the highest suicide mortality rates in the world for decades [4]. The numbers of suicide deaths have decreased overall between 2000 and 2022, although significantly more men (1 257 in 2022) than women (390 in 2022) suicide each year [5]. According to Eurostat data, Hungary has one of the highest suicide rates in the EU, with the Hungarian Southern Great Plain among the worst regions in the EU, with 23.91 suicide deaths per 100,000 inhabitants [6]. Despite high suicide rates in Europe, Hungary does not have a national suicide prevention strategy, action plan or central awareness-raising programmes [7, 8].

Although the number of publications on suicide increased 5.8 times between 1992 and 2021 [9] very few research has focused on public mental health aspects. Suicide is a complex and muticausal phenomenon, therefore it’s important that prevention includes community interventions [10, 11]. Research by Calear et al. [12] has drawn attention to the role of public help-seeking behaviour and intention, suicide stigma and suicide knowledge as key factors in the development of suicide prevention and community education programmes. A study by Cruwys et al. [13] found that suicide awareness was associated with more appropriate support recommendations, specifically for the person reporting suicidal thoughts. Suicide literacy seems to be a key factor which is linked to the concept of Mental Health Literacy (MHL) and it refers to community knowledge about mental disorders, as described by Jorm [14]. According to Jorm et al. [15], MHL has several components (e.g., recognition of types of mental disorder, knowledge of risk factors and causes, attitudes that facilitate recognition and appropriate help-seeking). Batterham et al. [16] argue that suicide literacy is part of mental health literacy, and the concept is found to be helpful assessing public knowledge related to suicidal thoughts and behaviours [17]. In line with that, Sharaf et al. [18] argued that misconceptions about suicide risk factors, treatments, and symptoms of suicide behavior may be at risk for suicide thoughts or behavior. Therefore is it valuable and important to compare populations on measures of suicide literacy [19]. In parallel to Calear et al. [12], there have been several recent publications on suicide stigma and suicide literacy [16, 17, 19–22], which, in addition to the Australian population sample, are also Turkish [23; 24; 25] Arabic [26, 27] Iranian [28] Persian [29] Bangla [30–32], Malay [33], German [34], Spanish [35, 36], Chinese [37] and from countries with high suicide rates, such as South Korea [38], Japan [39, 40] Nepal [41, 42] or Lithuania [43]. All studies used the Literacy of Suicide Scale (LOSS) created by Calear et al. [44].

While the number of studies assessing suicide literacy has increased significantly in recent years, little is known about European countries with high suicide rates such as Lithuania (21.3 deaths per 100 000 inhabitants) Hungary (17.1) or Slovenia (17.0) [6]. Therefore, we address the need for a comprehensive measure of suicide literacy in the Hungarian community. Although one study [45] measured suicide knowledge with the Revised Facts on Suicide Quiz and confirmed elevated misconceptions about suicide, the scale was not validated for the setting and did not cover critical aspects of suicide literacy. This is why we felt it important to adapt an inventory using the standard test adaptation methods according to the Test Adaptation Manual of the Hungarian Psychological Society. The LOSS scale includes 13 items from the RFOS but the RFOS was developed outside of the mental health literacy framework which means that it did not cover the prevention and treatment of suicidality [17]. There is also a tendency for published studies to use the short version (80%, vast majority of the studies, reported by Calear et al. [17]) of the LOSS [LOSS-SF], which contains 12 items, so that a number of important and relevant suicide knowledge is not assessed. As noted by Ludwig et al. [34] the majority of studies using LOSS are from Australia, with underrepresentation of data from European countries. The use of university-based samples and small sample size occur frequently [30, 42, 44, 46].

### Aim

The aim of the study is to translate and validate the Literacy of Suicide Scale (LOSS - long from) in Hungarian and examine suicide literacy among the Hungarian population.

## Methods

### Participants

The questionnaire was created with Google Forms and distributed on social media platforms with an access-based and snowball sampling procedure. Originally, 760 people applied to participate in the study on a voluntary basis, 2 people withdrew their consent, and 9 people were below the minimum age of 18 and hence were excluded. As a result, (N =) 749 responses were included in the study sample. The sample mean age was 32.4 years (SD: 14.5 years; range: 18–85 years). By gender, 382 respondents identified as female (51.0%), 364 identified as male (48.6%), and 3 identify as non-binary or other (0.4%); a total of 4 respondents reported identifying as transgender (0.5%).

### Ethical approval

The research project was approved by the Hungarian United Ethical Review Committee for Research in Psychology (EPKEB) (reference number: 2023 − 132). All procedures performed in this study were in accordance with the ethical standards of the institutional and/or national research committee and with the 1964 Helsinki declaration and its later amendments or comparable. Written Informed Consent was obtained from all subjects.

### Informed consent

Informed consent was obtained from all individual participants included in the study.

### Participant inducements and rewards

Participants were not offered any financial or equivalent inducements or rewards.

## Materials

### Socio-demographic questions

Socio-demographic questions explored gender identity, highest education, relationship status, and exposure to suicide, which are included in Table 1.

**Table 1:** Demographic characteristics of respondents split by gender identity (*N* = 749)

	Gender identity			
	Male *n* = 364 (48.6%)	Female *n* = 382 (51.0%)	Nonbinary *n* = 3 (0.4%)	Total *N* = 749
**Highest level of education**				
Elementary	13 (3.6%)	10 (2.6%)	–	23 (3.1%)
Secondary	212 (58.2%)	190 (49.7%)	2 (66.7%)	404 (53.9%)
Vocational	1 (0.3%)	6 (1.6%)	–	7 (0.9%)
Undergraduate	69 (19.0%)	95 (24.9%)	–	164 (21.9%)
Master’s Degree	61 (16.8%)	73 (19.1%)	1 (33.3%)	135 (18.0%)
Postgraduate	8 (2.2%)	8 (2.1%)	–	16 (2.1%)
**Exposure to suicide (*N* = 747**,** missing responses: 2)**				
Own suicide attempt	62 (17.0%)	83 (21.8%)	–	145 (19.4%)
Suicide attempt in one’s close circle	183 (50.4%)	228 (59.8%)	1 (33.3%)	412 (55.2%)
**Relationship status**				
In a relationship	169 (46.4%)	227 (59.4%)	2 (66.7%)	398 (53.1%)
Currently single	194 (53.3%)	153 (40.1%)	1 (33.3%)	348 (46.5%)
Other	1 (0.3%)	2 (0.5%)	–	3 (0.4%)
**Age**	33.4 (SD: 15.7)	31.4 (SD: 13.2)	25.0 (SD: 2.6)	32.4 (SD: 14.5)
**Age groups**				
Above average age	144 (60.4%)	151 (39.5%)	–	295 (39.4%)
Below average age	220 (39.6%)	231 (60.5%)	3 (100.0%)	454 (60.6%)

### Literacy of Suicide Scale (LOSS) questionnaire

The long version of LOSS was designed and assessed by Calear et al. [17, 44] in a university-based sample. The original scale has 26 items of a 3-option scale: ‘true’, ‘false’ or ‘I don’t know’. According to Batterham et al. [16], the LOSS items can be grouped into the following 4 domains (literacy themes): (a) *signs* (e.g., ‘People who talk about suicide rarely kill themselves’ [false]), (b) *causes/nature of suicidality* (e.g., ‘Those who attempt suicide do so only to manipulate others and attract attention to themselves’ [false]), (c) *risk factors* (e.g., ‘Most people who suicide are psychotic’ [false]), and (d) *treatment and prevention* (e.g., ‘People who have thoughts about suicide should not tell others about it’ [false]). The answers to the statements are scored based on the answer key assigning 1 point for each correct answer and 0 points to each incorrect or unsure answer. The total score can be calculated by summing up the points obtained in this way (percent correct).

We obtained the necessary permissions from the author for the Hungarian adaptation. The original version was translated into Hungarian by three independent translators in parallel, then a consensual Hungarian version was created by the main author. A back-translation of the consensual Hungarian version was created by the three independent translators, who then created a consensual back-translated English version. The consensual Hungarian and English versions were checked by an independent person and then approved by the author.

The LOSS questionnaire was designed following the modern (probability) test theory (IRT: item response theory) [16] with a two-parameter logistic model (2PL) using the weighted least squares estimator (WLSE) estimation method [17]. Accordingly, we used the related protocol [47, 48] for the psychometric evaluation of the Hungarian adaptation. IRT-based tests model the probability that an individual with a specific level of a latent trait (ability, aptitude) will respond correctly to a test item. This is expressed with the personal parameter (θ, theta) in a standardized form (mean 0, standard deviation 1). In the present case, this latent feature is suicide literacy as a skill. The two-parameter model assigns an item difficulty (b) and a discrimination (a) parameter to each item. Consequently, the higher the item difficulty value, the less likely it is that a respondent with a lower ability will give a correct answer. As a consequence, the item characteristic curve (ICC) of the item shifts to the right along the ability axis centered at θ = 0. The higher the discrimination value of the item, the more likely it is to separate respondents with lower and higher latent ability, and the steeper the characteristic curve.

### Statistical analysis

As a first step, we scored the raw answers based on the answer key: 0 points were given for incorrect or tentative (‘I don’t know’) answers, and 1 point for each correct answer. The statistical analysis was performed on the resulting dichotomous scale. Since the questionnaire evaluates only one latent ability, we had to verify the unidimensionality of the scale. Distributions in two-parameter dichotomous IRT tests do not follow the normal distribution, so we calculated the robust DETECT indicator [49] to examine unidimensionality. Values greater than 1.0 confirm strong multidimensionality, while values below 0.2 (also negative) confirm unidimensionality [50]. As a third step, we calculated the item difficulty, discrimination and personality parameters, as well as the fit indicators of each item: infit and outfit indicators, and the root mean square error of approximation for each item (RMSEA). While the item discrimination parameter can range from -∞ to +∞, [48] recommends that it should at least be positive (> 0) with a desirable range of 0.8 to 2.5, and any negative items to be discarded, while item difficulty is expected to fall between − 3 and + 3, although similarly, it can theoretically range from -∞ to +∞. The expected value of the infit and outfit indicators is 1.0, and the acceptable range, depending on the number of samples, is 1 ± 2/√N for infit and 1 ± 6/√N for outfit [48]. The limit of RMSEA values per item is 0.05 [51]. As a fourth step, due to the dichotomous 2PL method, the residual index of the standardized root mean square (SRMR), the mean of absolute deviations in observed and expected correlations (MADcor), the mean value of the absolute values of the Q3 indicator (MADQ3), the mean of absolute values of centered Q3 (MADaQ3). The cut-off values for the indicators are: MADcor values of up to 0.050 [52], SRMR values of up to 0.08 [29], MADQ3 of up to 0.20 and MADaQ3 of up to 0.10 [53]. As a fifth step, we examined the differential item functioning (DIF), that is, the bias of the items according to groups. The Mantel-Haenszel chi-square test [54] and the related ETS delta effect size test [55] were performed on sample mean age, gender identity (excluding nonbinary group due to extremely low number of responses, keeping only those identifying male or female) and own suicide attempt at a significance level of α = 0.01. Finally, to examine internal reliability, Cronbach’s alpha coefficient and McDonald’s omega were calculated with a limit of 0.7 [56]. Calculations related to IRT were performed in R [57]: the DETECT indicator and the model fit indicators with the sirt R package [58]; model parameters were calculated with the tam R package [59]. We used JASP [60] for additional analyses.

### Results

**Fig. 1:**
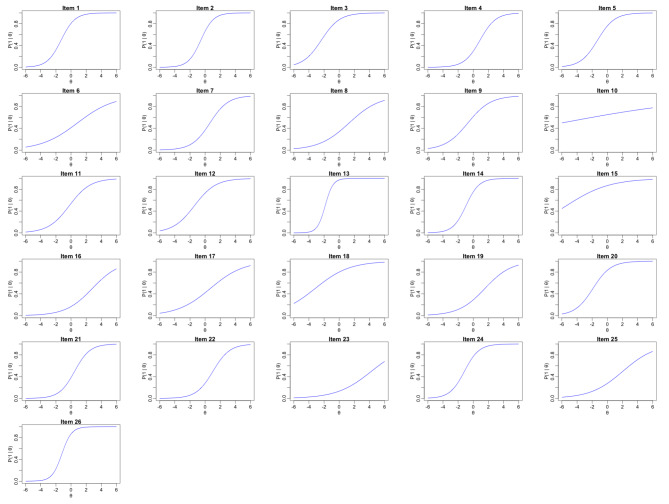
Characteristic curve of the LOSS-H items

The value of the DETECT indicator was − 0.47, so it can be concluded that the scale was unidimensional (i.e., items examine a single latent ability). Table 2 shows the discrimination (a) and item difficulty (b) values, as well as the item fit indicators. The individual characteristic curves are illustrated in Fig. 1.

**Table 2:** Item parameters and item fit indices

Item	Item parameters		Item fit		
	Discrimination value (a-value)	Item difficulty (b-value)	Infit	Outfit	RMSEA
1	1.092	-1.309	1.000	1.019	0.000
2	1.203	− 0.559	1.003	0.999	0.013
3	0.824	-2.350	1.011	0.980	0.000
4	0.964	0.901	0.996	1.003	0.029
5	0.892	-1.288	0.998	1.001	0.030
6	0.415	0.860	1.000	1.001	0.000
7	0.837	0.649	0.998	0.987	0.000
8	0.503	1.329	1.002	0.998	0.010
9	0.666	− 0.701	1.000	1.002	0.000
10	0.104	-5.960	1.000	1.000	0.000
11	0.721	− 0.226	0.999	0.999	0.005
12	0.711	-1.407	0.999	1.006	0.000
13	1.938	-1.847	1.013	0.976	0.000
14	1.163	− 0.956	1.000	0.976	0.011
15	0.351	-5.394	0.998	1.002	0.009
16	0.570	2.817	1.000	0.992	0.025
17	0.458	0.697	0.998	0.997	0.000
18	0.439	-3.164	0.999	0.998	0.036
19	0.578	1.548	1.004	0.985	0.031
20	0.849	-1.894	1.008	0.963	0.027
21	0.992	0.479	1.006	0.986	0.017
22	0.908	1.043	0.998	1.010	0.000
23	0.437	4.278	0.996	1.004	0.010
24	1.040	-1.160	0.990	1.040	0.018
25	0.469	2.063	1.003	0.997	0.000
26	1.557	-1.213	0.991	0.976	0.000

Model fit indices met acceptability criteria: SRMR = 0.046, MADcor = 0.046, MADQ3 = 0.060, MADaQ3 = 0.055. The internal reliability indicators were adequate: Cronbach α = 0.722, McDonald ω = 0.716.

All items have appropriate infit and outfit and item-RMSEA values. The infit values were between 0.927 and 1.073, and the outfit values were between 0.781 and 1.219, meaning all items met the acceptability criteria. The RMSEA values for each item were also below the upper limit of 0.05.

The test items presented a diverse range of discrimination (a) and difficulty (b) parameters. The discrimination (a) values varied from 0.104 to 1.938, with most items falling within the generally acceptable range of 0.0 to 2.0 as suggested by de Ayala [48]. This range indicates that the items had varying degrees of effectiveness in distinguishing between individuals with different levels of the latent trait being measured. The lowest discrimination value was 0.104 (item 10), which might suggest a potential lack of effectiveness in distinguishing between different ability levels, however it did not fall below zero, and didn’t have to be discarded automatically. The highest discrimination value was 1.938 (item 13), suggesting that this item was highly effective in differentiating between test-takers with varying abilities.

The item difficulty (b) parameters were between − 3 and + 3, indicating a balanced mix of item difficulties suitable for assessing a broad spectrum of ability levels. However, some items had difficulty parameters outside this range. Items with difficulty values outside the range could either be too easy or too hard for the test-takers. One item (23) was considerably more difficult than expected (b = 4.278), with a relatively low but still acceptable discrimination parameter value (a = 0.437). Three items (10, 15 and 18) had a low item difficulty beyond the desired range (-5.960, -5.394 and − 3.164 respectively). Items 10, 15, and 18, with their low difficulty parameters, could indicate a need for further evaluation. However, these items were not removed as the overall model fit metrics did not improve upon their exclusion. Despite some outliers, the test effectively measures a broad spectrum of abilities and maintains overall reliability.

We found statistically significant non-invariances with large effect sizes with several items as summarized in Table 3. Differential item functioning (DIF) occurs when individuals from different groups (e.g., based on age, gender, or own suicide attempt) with the same underlying ability have a different probability of responding correctly to a particular item [61]. DIF indicates potential bias in test items, where an item may be easier or harder for one group compared to another, even when their overall ability levels are equivalent.

As expected, item 16 (age-based risk) had a statistically significant non-invariance based by age groups; item 17 (gender-based risk) had large non-invariance by gender groups; and items pertaining to mentalisation of those who suicide (7, 12, 20) had statistically significant non-invariance for those who reported past suicidality. In addition, item 17 and items 4, 5, relating to mental health and healthcare systems, also showed statistically significant non-invariance by age group. We found statistically significant item distortion for item 14 (‘Very few people have thoughts about suicide.’) and in item 26 (‘Those who attempt suicide do so only to manipulate others and attract attention to themselves’) by gender. This means we must be cautious when comparing means and interpreting results across age and gender groups for these specific items.

**Table 3:** Indicators of differential item functioning (DIF)

Item	By age			By gender identity			By own suicide attempt		
	Distortion		Effect size	Distortion		Effect size	Distortion		Effect size
	𝜒²_MH_	*p*	Δ_MH_	𝜒²_MH_	*p*	Δ_MH_	𝜒²_MH_	*p*	Δ_MH_
1	0.071	0.790	0.927	0.015	0.904	0.097	1.281	0.258	− 0.730
2	0.083	0.774	1.073	1.081	0.299	0.468	0.446	0.504	− 0.396
3	4.197	0.040	0.588	2.208	0.137	− 0.826	5.304	0.021	-1.530
4	18.531	< 0.001	0.441	0.289	0.591	0.251	0.645	0.422	− 0.472
5	12.427	< 0.001	1.962	1.202	0.273	− 0.501	2.699	0.100	1.032
6	2.716	0.099	1.337	0.155	0.694	− 0.177	12.033	0.001	1.667
**7**	1.664	0.197	0.784	13.953	< 0.001	-1.492	15.297	< 0.001	1.845
8	1.879	0.170	1.289	0.706	0.401	0.358	0.045	0.832	0.150
9	7.024	0.008	1.586	0.122	0.727	0.161	5.449	0.020	-1.125
10	5.768	0.016	0.657	1.962	0.161	− 0.554	0.020	0.888	− 0.114
11	0.018	0.892	0.965	0.000	0.984	− 0.036	2.449	0.118	− 0.762
12	0.000	0.983	0.979	1.771	0.183	0.588	9.341	0.002	1.896
13	0.021	0.886	0.995	0.568	0.451	− 0.648	0.050	0.824	− 0.394
14	0.348	0.556	1.143	18.333	< 0.001	-1.965	2.553	0.110	0.964
15	3.608	0.058	1.611	2.593	0.107	0.947	1.841	0.175	1.134
16	10.960	< 0.001	0.485	0.255	0.614	− 0.291	0.662	0.416	− 0.602
17	22.921	< 0.001	2.334	29.600	< 0.001	2.105	2.377	0.123	− 0.792
18	2.763	0.096	0.695	8.002	0.005	1.345	0.010	0.920	0.134
19	0.600	0.438	0.859	3.732	0.053	− 0.801	0.833	0.361	− 0.504
20	8.397	0.004	0.534	0.041	0.839	0.136	21.656	< 0.001	-2.605
21	2.870	0.090	1.371	2.328	0.127	0.630	3.555	0.059	− 0.979
22	7.743	0.005	0.592	4.438	0.035	0.892	2.079	0.149	− 0.826
23	0.003	0.956	0.960	3.091	0.079	− 0.982	0.225	0.636	0.371
24	0.683	0.408	1.190	4.642	0.031	− 0.999	1.812	0.178	0.889
25	0.015	0.903	1.039	9.521	0.002	1.282	1.990	0.158	0.740
26	1.969	0.161	1.398	10.444	0.001	-1.629	2.693	0.101	1.238

The distortion indicator is the Mantel-Haenszel chi-square with *p* significance, the effect size indicator is the ETS delta

In our sample, the sample mean score was 14.59 (SD = 4.05), where 473 (63.15%) respondents scored more than 13 points out of a maximum of 26, however there is no cutoff or center point defined for our scale. The proportion of correct answers per item is presented in the supplementary material. Respondents younger than the sample mean age (M = 15.30, SD = 3.49) had a higher total score than older respondents (M = 13.52, SD = 4.59), but a comparison between age groups should be interpreted with caution, due to DIF items.

## Discussion

The present study focused on validating the long-form version of LOSS in Hungary. Our results indicated a good model fit, acceptable internal reliability and appropriate item characteristics. This suggests that the Hungarian version of the Suicide Literacy scale with its 26 items can be used for assessing suicide literacy in the Hungarian population. Few studies have used [17, 21] or adapted the 26-item long version of LOSS, particularly relying on IRT. Iranian researchers developed a final model with only 25 questions using regular confirmatory factor analysis [28]. Phoa et al. [33] used the Rasch model to derive a 26-item Malaysian version of the scale, and they found a weak but significant negative correlation between age and suicide literacy, but no correlation was found between mental health and suicide literacy. The Malay version of the LOSS scale was found to be unidimensional. Turkish researchers [23] adapted the LOSS scale on a sample of Turkish university students based on Item Response Theory confirming unidimensionality in their version as well.

The Hungarian version of the LOSS model fit indices met acceptability criteria: SRMR = 0.046, MADcor = 0.046, MADQ3 = 0.060, MADaQ3 = 0.055. The internal reliability indicators were adequate: Cronbach α = 0.722, McDonald ω = 0.716. In the Persian version of the LOSS, Jafari et al. [28] reported the value of 0.866 for McDonald ω, and 0.859 for Cronbach α. Chan et al. [21] reported 0.71 for Cronbach α in the study amongst Australian medical students, using the long form of the LOSS.

The size of the sample qualifies in the large range compared to other studies that have also validated the long version of the LOSS scale [36]. A sample of 749 respondents were included in the study sample. Calear et al. [17] analysed the data of 466 people, while Jafari et al. [28] used the sample of 954 participants. The research by Öztürk & Akin [23] was conducted with 1100 university students, while Phoa et al. [33] recruited 750 (study 1) and 867 (study 2) respondents across West Malaysia. Kennedy et al. [19] in the rural areas of Australia utilized a sample of 536 people. In our sample, gender distribution was balanced (382 women, 364 men, and three non-binary individuals), while other studies were gender-biased in many cases [19, 26, 36; 28, 17]. Our study established an age range from 18 to 85, the sample mean age was 32.4 years (SD: 14.5 years). The sample features a unique wide range of ages than other studies [16 17, 36]. In our sample, 63.15% of the respondents scored higher than a theoretical midpoint (13 points) which is comparable with studies in countries like Australia: in the development and validation samples 63.4% scored higher than 13 points among the university sample, and 58.2% among the community sample [17], Phoa et al. [33] reported 54.0%. This is purely a nominal comparison of ratios between countries; however, no official cutoff point was designated or should be assigned for this literacy scale.

The highest discrimination value was perceived in item 13 (People who have thoughts about suicide should not tell others about it - false), suggesting that this item was highly effective in differentiating between test-takers with varying abilities. It is a noteworthy finding given the fact that non-disclosure is a substantial problem in adults experiencing suicidal ideation [62], and among acute psychiatric inpatients [63]. On the other hand, a study by Nicholas et al. [64] considered important the belief in the myth that ‘asking someone about suicide could make them start thinking about it’ suggesting that it may have the greatest effects on helping behaviour.

Item 23 (A time of high suicide risk in depression is at the time when the person begins to improve - true) was considerably more difficult than other items. Three items, all belonging to the risk factors domain (item 10: A person who has made a past suicide attempt is more likely to attempt suicide again than someone who has never attempted –true, item 15: People who are anxious or agitated have a higher risk of suicide and – true, item 18: People with relationship problems or financial problems have a higher risk of suicide – true), had low item difficulty.

We found that suicide literacy levels decreased with age in our sample. However, this should be assessed with caution as some items by age group have differential item functioning. Ludwig et al. [34] found that suicide literacy was negatively related to age, while positively related to education. Japanese researchers [39] measured significantly higher suicide stigma values and lower levels of suicide literacy compared to Australian and German participants. In their study, younger age, lower education, greater exposure to suicide, and greater social support were related to greater suicide literacy. According to Collado et al. [36] it is necessary to strengthen the general population’s understanding of suicide, in terms of causes/nature of suicidality, risk factors, signs and prevention. A longitudinal study found that adequate health literacy may serve as an important protective factor for poor long-term mental health outcomes among adolescents [65].

In terms of suicide crisis and risk factors, Erdos & Javor [66] discussed the strong social and cultural factors in the background of suicide in Hungary, and the approach of discursive suicidology [67] which states that Hungarian is a metaphorical language. Specific cultural or language factors may also influence suicide prevention in Hungary. Ambivalence and cry-for-help communication [68], playing a key role in suicide risk recognition. Overt requests for help to covert, barely perceptible, often unconscious signals, or even vague hints, gradual changes in behaviour make recognition difficult [68, 69]. It is important to emphasize that suicide risk recognition and suicide prevention is not only a medical-psychiatric task, therefore it is important to create a suicide prevention literacy improvement programme targeting both the civil population and health professionals. According to Rihmer [70], education of physicians, health professionals and the general public is effective in reducing suicide mortality. H-LOSS provides an opportunity for research not only in the community, but also with health workers (doctors, nurses), young people (adolescent population), older adults, mental health professionals (psychologists, psychiatrists), and teachers. Each member of the society has a specific competence, opportunity, responsibility and role in suicide prevention [71].

### Limitations

Our validation study used convenience sampling, with a cross-sectional, online questionnaire design, so it may not be considered representative of the Hungarian population and it is not implausible that participants had a higher level of interest and literacy already compared to the general population. Our research did not investigate the role of suicide literacy on help-seeking or on suicide-related stigma, as we focused on statistical validation. Other studies draw attention to the possible association between low suicide knowledge and suicide stigma/shame, therefore we consider it important to adapt the Stigma of Suicide Scale (SOSS) and compare it with H-LOSS. The concept of suicide literacy like the concept of MHL assumes the superiority of expert psychiatric knowledge over lay beliefs [14], therefore incongruities produced by operationalisation of suicide literacy may preserve structural inequities in epistemic practice [72].

## Conclusions

Overall, the H-LOSS is an adequate scale for assessing public knowledge of suicide in population-based studies and can be used to assess suicide literacy, identifying knowledge gaps about suicide, The scale may be used to inform mental health promotion and community education interventions, evaluate the effectiveness of interventions to improve suicide literacy, and conduct cross-cultural comparative studies.

### Electronic supplementary material

Below is the link to the electronic supplementary material.

Supplementary Material 1

## Data Availability

The data that support the findings of this study are available from the corresponding author upon reasonable request.

## Abbreviations

IRT: Item response theory
WLSE: Weighted least squares estimator
2PL: Two-parameter logistic model
LOSS: Literacy of suicide scale
MHL: Mental health literacy
H-LOSS: Hungarian version of the literacy of suicide scale
SRMR: Standardized root mean square
RMSEA: Square error of approximation
