# Supplementary material for: Myths about suicide - validating the Hungarian version of the Literacy of Suicide Scale (H-LOSS) on a community sample
Source: BMC Public Health. 2024 Aug 29;24:2351. doi: 10.1186/s12889-024-19841-8 (PMC11360470; doi:10.1186/s12889-024-19841-8)
Supplement: Supplementary file 1 — Supplementary Material 1 [file 12889_2024_19841_MOESM1_ESM.docx]

**Supplementary Table S1: **The proportion of correct answers to H-LOSS****

|  |  | Correct answers | |
| --- | --- | --- | --- |
| Item in Hungarian | Topic | N | Relative frequency |
| 1. Semmit sem lehet tenni annak érdekében, hogy megakadályozzunk egy öngyilkossági kísérletet, ha az illető már eldöntötte, hogy megöli magát. (*Hamis*) | Treatment and prevention | 571 | 76.2% |
| 2. Egy pszichiáter depressziót diagnosztizálna minden öngyilkosságot elkövető személynél. (*Hamis*) | Causes/nature of suicidality | 471 | 62.9% |
| 3. Egy pszichiáter vagy pszichológus felkeresése segíthet az öngyilkosság megelőzésében. (*Igaz*) | Treatment and prevention | 635 | 84.8% |
| 4. A legtöbb öngyilkosságot elkövető ember pszichotikus (mentális problémákkal küzd). (*Hamis*) | Risk factors | 244 | 32.6% |
| 5. Csak hozzáértő szakemberek tudnak segíteni azokon az embereken, akik öngyilkosok akarnak lenni. (*Hamis*) | Treatment and prevention | 546 | 72.9% |
| 6. Az alkoholizmus és az öngyilkosság között jelentős összefüggés van. (*Igaz*) | Risk factors | 311 | 41.5% |
| 7. Azok az emberek, akik beszélnek az öngyilkosságról, ritkán ölik meg magukat. (*Hamis*) | Signs | 288 | 38.5% |
| 8. Azok az emberek, akik öngyilkosságot akarnak megkísérelni, könnyen meggondolhatják magukat. (*Igaz*) | Signs | 260 | 34.7% |
| 9. Az öngyilkosságról való beszéd minden esetben megnöveli az öngyilkosság kockázatát. (*Hamis*) | Causes/nature of suicidality | 453 | 60.5% |
| 10. Egy olyan személy, akinek már volt öngyilkossági kísérlete, hajlamosabb újra megkísérelni az öngyilkosságot, mint egy olyan személy, aki még sosem akart végezni magával. (*Igaz*) | Risk factors | 487 | 65% |
| 11. A média öngyilkossággal kapcsolatos hírközlései elkerülhetetlenül bátorítani fogják az embereket az öngyilkosságra. (*Hamis*) | Causes/nature of suicidality | 402 | 53.7% |
| 12. Nem minden esetben tervezi meg előre az elkövető az öngyilkosságot. (*Igaz*) | Signs | 533 | 71.2% |
| 13. Akiknek öngyilkossági gondolataik vannak, nem kellene beszélniük erről másoknak. (*Hamis*) | Treatment and prevention | 684 | 91.3% |
| 14. Nagyon kevés embernek vannak öngyilkossági gondolatai. (*Hamis*) | Causes/nature of suicidality | 530 | 70.8% |
| 15. A szorongó vagy zaklatott emberek hajlamosabbak öngyilkosság elkövetésre. (*Igaz*) | Risk factors | 647 | 86.4% |
| 16. A legtöbb öngyilkosságot elkövető személy 30 évnél fiatalabb. (*Hamis*) | Risk factors | 136 | 18.2% |
| 17. A férfiak hajlamosabbak az öngyilkosságra, mint a nők. (*Igaz*) | Risk factors | 318 | 42.5% |
| 18. (Pár)kapcsolati vagy anyagi problémákkal küzdő emberek esetében nagyobb az öngyilkosság elkövetésének kockázata. (*Igaz*) | Risk factors | 593 | 79.2% |
| 19. A legtöbb öngyilkosságot elkövető ember nem tervez a jövőre. (*Hamis*) | Signs | 227 | 30.3% |
| 20. Ha közvetlenül feltennénk valakinek a kérdést, hogy „Meg szeretnéd ölni magad?”, akkor ez a kérdés nagy valószínűséggel ahhoz fog vezetni, hogy a személy öngyilkosságot kísérel meg. (*Hamis*) | Causes/nature of suicidality | 602 | 80.4% |
| 21. Egy öngyilkosságra hajlamos személy mindig öngyilkosságra hajlamos marad, és foglalkoztatni fogja az öngyilkosság gondolata. (*Hamis*) | Causes/nature of suicidality | 302 | 40.3% |
| 22. Aki öngyilkosság követ el, az mentális problémákkal küzd. (*Hamis*) | Causes/nature of suicidality | 231 | 30.8% |
| 23. Egy depressziós ember esetében akkor magas az öngyilkosság kockázata, amikor állapota javulni kezd. (*Igaz*) | Signs | 106 | 14.2% |
| 24. Az öngyilkosság indítékai és okai könnyen megállapíthatóak. (*Hamis*) | Causes/nature of suicidality | 547 | 73.0% |
| 25. A legtöbb öngyilkosságot megkísérlő embernek nem sikerül megölnie magát. (*Igaz*) | Causes/nature of suicidality | 213 | 28.4% |
| 26. Azok, akik öngyilkosságot kísérelnek meg, csak azért teszik, hogy manipuláljanak másokat, és felhívják magukra a figyelmet. (*Hamis*) | Causes/nature of suicidality | 594 | 79.3% |
